# Hypercoagulability in Patients with Non-Alcoholic Fatty Liver Disease (NAFLD): Causes and Consequences

**DOI:** 10.3390/biomedicines10020249

**Published:** 2022-01-24

**Authors:** Armando Tripodi, Rosa Lombardi, Massimo Primignani, Vincenzo La Mura, Flora Peyvandi, Anna L. Fracanzani

**Affiliations:** 1 Fondazione IRCCS Ca’ Granda Ospedale Maggiore Policlinico, Angelo Bianchi Bonomi Hemophilia and Thrombosis Center and Fondazione Luigi Villa, 20122 Milan, Italy; vincenzo.lamura@unimi.it (V.L.M.); flora.peyvandi@unimi.it (F.P.); 2 Fondazione IRCCS Ca’ Granda Ospedale Maggiore Policlinico, Unit of Internal Medicine and Metabolic Disease, 20122 Milan, Italy; rosa.lombardi@unimi.it (R.L.); anna.fracanzani@unimi.it (A.L.F.); 3 Department of Pathophysiology and Transplantation, Università degli Studi di Milano, 20122 Milan, Italy; 4 Fondazione IRCCS Ca’ Granda Ospedale Maggiore Policlinico, First Division of Gastroenterology, 20122 Milan, Italy; massimo.primignani@policlinico.mi.it

**Keywords:** procoagulant imbalance, thrombin generation, antithrombin, protein C, factor VIII, thrombomodulin

## Abstract

Non-alcoholic fatty liver disease (NAFLD) is the most common chronic liver disease, and it is anticipated that it could become even more prevalent in parallel with an increase in the incidence of metabolic diseases closely related to NAFLD, such as obesity, type II diabetes, dyslipidemia, and arterial hypertension. In addition to liver impairment, NAFLD is associated with cardiovascular diseases. Fibrosis, atherosclerosis, and venous thrombosis are basically the pathogenic mechanisms behind these clinical manifestations, and all are plausibly associated with hypercoagulability that may, in turn, develop because of an imbalance of pro- vs. anticoagulants and the presence of such procoagulant molecular species as microvesicles, neutrophil extracellular traps (NETs), and inflammation. The assessment of hypercoagulability by means of thrombin generation is a global procedure that mimics the coagulation process occurring in vivo much better than any other coagulation test, and is considered to be the best candidate laboratory tool for assessing, with a single procedure, the balance of coagulation in NAFLD. In addition to defining the state of hypercoagulability, the assessment of thrombin generation could also be used to investigate, in clinical trials, the best approach (therapeutic and/or lifestyle changes) for minimizing hypercoagulability and, hence, the risk of cardiovascular diseases, progression to atherosclerosis, and liver fibrosis in patients with NAFLD.

## 1. Introduction

**Figure 1:**
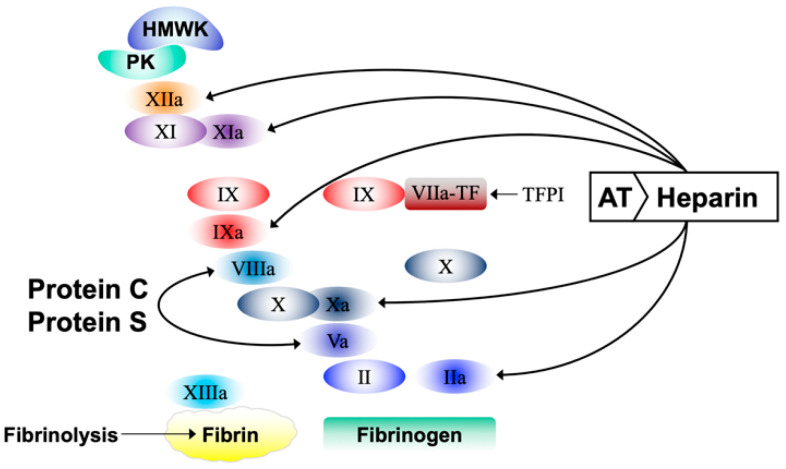
Schematic representation of the iterative coagulation activation starting from the complex TF-FVIIa formation and leading to thrombin (IIa) generation and fibrin formation. Among the other functions (not shown in the scheme), thrombin activates FXI, FXIII, FV, FVIII, and protein C (see also Figure 3). Arrows refer to factors inhibited by the antithrombin–heparin complex or by the activated protein C–protein S complex or TFPI. The suffix “a” denotes activated coagulation factors. HMWK, high molecular weight kininogen; PK, prekallicrein; Roman numbers refer to coagulation factors; TFPI, tissue factor pathway inhibitor; AT, antithrombin; TF, tissue factor.

Coagulation is a tightly regulated and complex humoral/cellular mechanism, which in normal conditions allows blood fluidity within the vascular system but helps to stop bleeding at the site of a blood vessel wall injury. The main components of coagulation are the procoagulant and the anticoagulant factors. The procoagulants start their action soon after the formation of a complex between tissue factor and plasma factor VIIa at the site of a vessel wall injury, when tissue factor that is normally hidden into the cell membranes meets circulating blood and starts a series of iterative activations leading to thrombin generation and, ultimately, to the conversion of fibrinogen to fibrin (Figure 1). Procoagulants are physiologically contrasted by the naturally occurring anticoagulants (i.e., antithrombin, protein C, protein S, and the tissue factor pathway inhibitor) (Figure 1). In normal conditions the pro- and anticoagulants are perfectly balanced, and therefore, unwanted thrombin generation and fibrin deposition do not occur. However, there may be clinical conditions in which the balance between the two drivers is perturbed to such an extent that it results in a state of hypo- or hypercoagulability, which may be associated with hemorrhage or thrombosis, respectively. Non-alcoholic fatty liver disease (NAFLD) is the most common chronic liver disease, which beside liver impairment, is associated with an increased risk of cardiovascular diseases and atherosclerosis. Because of this, NAFLD should be the prototype of the diseases to be investigated for coagulation abnormalities. In this article, we aim to review the coagulation derangement associated with NAFLD, with special interest on hypercoagulability investigated by means of the last generation of coagulation tests.

## 2. Hypercoagulability

Hypercoagulability is defined as a procoagulant imbalance due to increased levels of procoagulants, decreased levels of anticoagulants, or both. Other circumstances where hypercoagulability may occur in the absence of apparent coagulation derangement is the presence of one of the following: (i) Elevated circulating microvesicles that stem from monocytes, platelets, or endothelial cells, which upon activation release, into the circulation, massive amounts of small cytoplasmic vesicles possessing, on their membranes, the procoagulant asset of the parent cells (e.g., tissue factor from monocytes, negatively charged phosphatidylserine from platelets, etc.), which is, therefore, disseminated into the systemic circulation [1]. This may dramatically increase the state of plasma hypercoagulability, as shown by the increased risk of cardiovascular events [2] and venous thromboembolism associated with elevated levels of microvesicles [3]. (ii) Ongoing inflammation, which through the massive release of proinflammatory cytokines perturbs the balance between pro- and anticoagulants leading to hypercoagulability [4]. (iii) Elevated circulating chromatin substances, collectively known as neutrophil extracellular traps (NETs), which are the hallmark of heightened neutrophil activation [5]. NETs support, on their surface, the activation of coagulation factors and are considered to be risk factors for venous thromboembolism [6,7].

### How to Assess Hypercoagulability

In principle, hypercoagulability can be assessed by performing basic coagulation tests such as: (i) the prothrombin and activated partial thromboplastin time (PT and APTT); (ii) the measurement of individual procoagulants, or (iii) the measurement of individual naturally occurring anticoagulants (i.e., antithrombin, protein C, protein S, or tissue factor pathway inhibitor). However, none of these approaches, if used as standalone laboratory tools, are suitable to account for the complex interaction between the pro- and anticoagulant drivers operating in vivo. PT and APTT are global coagulation tests, responsive to most of the procoagulants (they are, in fact, abnormally prolonged in congenital hemorrhagic deficiencies such as hemophilia and other congenital hemorrhagic disorders). PT and APTT are, however, not equally responsive to the action of naturally occurring anticoagulants [8]. They are, in fact, normal in patients with congenital deficiency of antithrombin, protein C, or protein S conditions where they should be shortened, since carrier patients generate much more thrombin than healthy subjects. Hence, on the one hand, PT and APTT are unfit to represent the process of coagulation occurring in vivo. On the other hand, naturally occurring anticoagulants, if measured as standalone parameters, although useful for identifying hypercoagulability in patients with congenital deficiency, would not account for the balance represented by the presence of their counterpart (i.e., the procoagulants) that could be variably increased or decreased depending on the circumstances, especially in acquired coagulopathies. Finally, none of the above approaches account for the contribution to hypercoagulability provided by the presence of inflammation, high procoagulant microvesicles, or NETs. In contrast, hypercoagulability can be conveniently investigated by the last generation of global coagulation procedures, called thrombin generation, which are much more representative of the coagulation process that occurs in vivo than the basic tests, i.e., PT, APTT, and individual pro- or anticoagulants, when taken as standalone measurements.

## 3. Thrombin Generation Procedures

**Figure 2:**
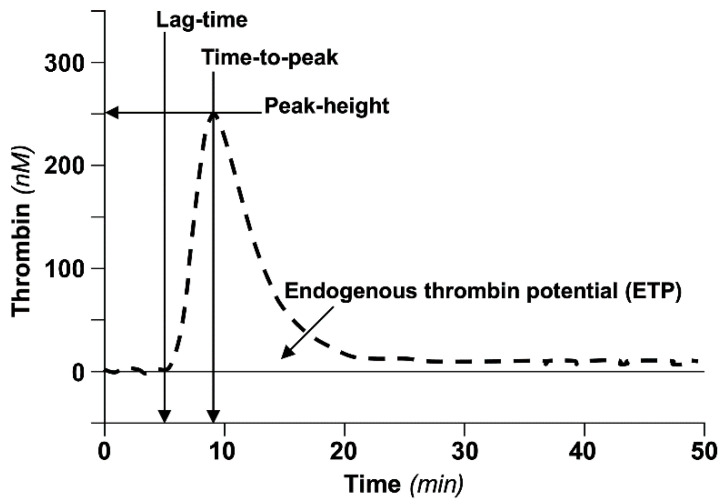
Schematic representation of the thrombin generation curve (thrombogram) with relevant parameters.

Thrombin generation defines a global coagulation procedure that, upon in vitro activation of coagulation in platelet-poor plasma by small amounts of tissue factor and negatively charged synthetic phospholipids (triggers), has been designed to continuously monitor the generation of thrombin [9]. The quality and quantity of triggers have been chosen to mimic as much as possible the conditions operating in vivo [10]. The procedure can be run in a completely automated fashion and the system constructs a thrombin generation curve (thrombogram), characterized by an ascending arm, which records the thrombin concentration generated as a function of the action of the procoagulants and a descending arm, which records the thrombin neutralized by the action of the naturally occurring anticoagulants (Figure 2).

**Figure 3:**
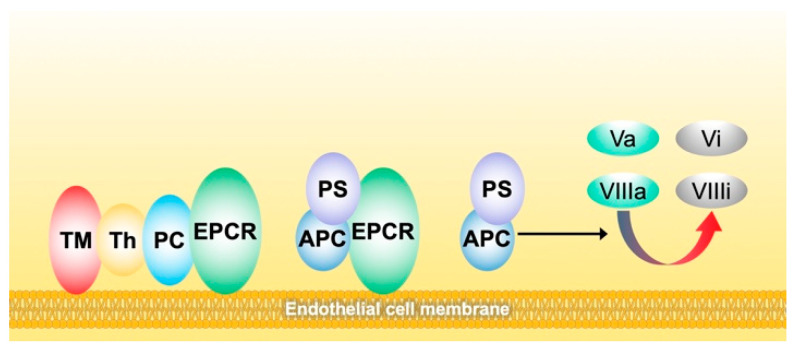
Schematic representation of the activation of protein C on the membrane of endothelial cells and the mechanism of action of the complex of activated protein C/protein S in inhibiting factor Va and factor VIIIa. Instrumental to the mechanism are two endothelial receptors, thrombomodulin (TM) that binds thrombin (Th) and endothelial protein C receptor (EPCR) that binds plasma protein C (PC). The proximity of the above receptors localizes the conversion of the substrate (PC) by the enzyme (Th) into activated PC (APC). APC in complex with its plasmatic cofactor protein S (PS) eventually inhibits factors Va and VIIIa, thus, downregulating thrombin production.

The main parameters of the thrombogram are (i) the lag time, defined as the time needed for the first amounts of thrombin to appear; (ii) the time-to-peak, defined as the time needed to reach the peak thrombin; (iii) the peak thrombin, defined as the greatest amount of generated thrombin; (iv) the area under the curve, called endogenous thrombin potential (ETP), which accounts for the net amount of thrombin that can be generated under the driving forces of the procoagulants opposed by the anticoagulants operating in plasma. Prolonged or short lag time and time-to-peak can be considered to be indexes of hypo- or hypercoagulability, respectively. Conversely, low or high peak thrombin and ETP can be considered to be indexes of hypo- or hypercoagulability, respectively. The above parameters can be recorded in the presence or absence of exogenously added thrombomodulin (TM) [11]. TM is the physiological activator of protein C, which is, in turn, the physiological inhibitor to factor VIIIa and factor Va (Figure 3).

TM is, however, located on endothelial cells and much less in plasma, and therefore, any assay meant to assess coagulation in plasma (namely PT, APTT, or protein C/protein S) cannot account for the optimal activation of protein C and, hence, for the inhibition of factor VIIIa, both representing potent regulators of thrombin generation. Hence, the evaluation of ETP measured in the presence or absence of TM or as the ratio between the two values (called the ETP-TM ratio) can be considered to be the best in vitro representation of the coagulation process operating in vivo [11]. In principle, the ETP-TM ratio represents an index of the resistance of plasma to the anticoagulant action of TM, which, in turn, depends on the imbalance between factor VIII and protein C, the two major regulators of thrombin generation. Consequently, the ETP-TM ratio is a reliable index of plasma hypercoagulability (the higher the ratio the greater the hypercoagulability) [11].

## 4. General Features of NAFLD

Hypercoagulability is one of the three components identified by Virchow (i.e, hypercoagulability, endothelial dysfunction, and reduced/turbulent blood flow) that, alone or in combination, can trigger venous thromboembolism. Interestingly, hypercoagulability is very often associated with clinical conditions characterized by an increased risk of cardiovascular diseases, either arterial or venous, or both. The above clinical conditions include type II diabetes [12], obesity [13], liver cirrhosis [14], Cushing disease [15], inflammatory bowel diseases [16,17], and others. Owing to its clinical characteristics (see below), non-alcoholic fatty liver disease (NAFLD) could be the prototype of the composite diseases that could be investigated for hypercoagulability.

NAFLD is a common clinical condition with an estimated prevalence of 23% in Europe, 24% in the USA, 32% in the Middle East, and 27% in Asia [18]. Recent trend analyses have shown that, in the period 1991–2019, NAFLD increased from 21.9 to 37.3% (yearly increase of 0.7% (*p* < 0.0001)) [19]. NAFLD is relatively more common in men with advancing age but is also observed in obese children and adolescents with sequels similar to those observed in adults [20,21]; in women, the prevalence of NAFLD becomes similar to that of men after menopause [22]. NAFLD is a significant global public health burden to health care systems across the world and is expected to increase along with the incidence of such metabolic derangements typically observed in patients with type II diabetes, obesity, or dyslipidemia. Furthermore, being the most common liver disease, NAFLD is expected to be the leading cause of liver transplantation in the near future [23].

NAFLD ranges from simple steatosis, defined by an accumulation of fat greater than 5% of liver weight, to the progressive form of non-alcoholic steatohepatitis (NASH), whose hallmark is inflammation and hepatocellular death that can progress to fibrosis, up to the most severe form of metabolic cirrhosis. Interestingly, this life-threatening progression of liver disease severity has been associated with an increased risk of atherosclerosis (i.e., coronary artery and abdominal aortic calcification and the presence and progression of carotid intima-media thickness [24,25], as well as high risk of cardiovascular diseases [26], the latter accounting for the higher rate of total mortality observed in NAFLD as compared with the general population. NAFLD has also been associated with the occurrence of venous thromboembolism (including deep vein thrombosis of the lower limbs, pulmonary embolism, and splanchnic vein thrombosis) [27,28]. Hypercoagulability is considered to be a risk factor that alone or (more likely) in combination with other genetic (e.g., prothrombotic polymorphisms) and/or circumstantial risk factors (e.g., old age, cancer, recent surgery, metabolic syndrome, oral contraceptive intake, and/or hormonal replacement therapy, etc.) may increase the risk of arterial and venous thrombotic complications. In this respect, the general features and clinical characteristics of NAFLD make it a prototype to look at regarding an association with hypercoagulability as a cause for the cardiovascular risk in metabolic diseases.

Environmental and genetic factors contribute to the pathogenesis of NAFLD that, in addition to being a very prevalent disease in the general population, is burdened by a series of complications, not only hepatic, which determine morbidity and mortality through multiple mechanisms not yet completely understood. For example, in addition to liver diseases, type II diabetes, which is per se a risk factor of cardiovascular diseases, is frequently associated with NAFLD [29] and may increase, by two times, the risk of developing NASH and severe liver-related complications (cirrhosis, liver failure, and hepatocellular carcinoma). In addition, patients with established type II diabetes also show a high prevalence of fatty liver. All in all, type II diabetes and NAFLD are closely associated, as they share several cardiometabolic risk factors and their clinical evolution is strongly influenced by each other. The prevalence of imaging-defined NAFLD in diabetes is about 60–75%, and that of liver fibrosis, which in NAFLD has been defined as the strongest predictor of long-term adverse clinical outcomes, is about 5–20% [30]. Interestingly, hypercoagulability detected by thrombin generation has been shown in patients with type II diabetes and seems to be mediated by increased factor VIII and the presence of procoagulant microvesicles [12]. Other relevant extrahepatic manifestations in NAFLD are neoplasms of the gastrointestinal tract [31,32] and chronic kidney disease [33], which share the same risk factors as type II diabetes, as well as metabolic syndrome, obesity, hypertension, and obstructive sleep apnea [34], even in the absence of obesity or metabolic syndrome and hypothyroidism [35], which are associated with a higher risk for overall and cardiovascular mortality, polycystic ovary syndrome [36], and psoriasis [37]. Many risk factors influence survival in NAFLD/NASH, whose leading causes of death are cardiovascular diseases. A similar association has been observed for liver transplant outcome. Indeed, liver transplant recipients with NASH have significantly more fatal and nonfatal post-transplant cardiovascular events than recipients without NASH [38,39].

Among the above, the relatively high prevalence of cardiovascular diseases is the most relevant for this review and will be taken into consideration in this article. The proposed mechanisms for the development of cardiovascular diseases in patients with NAFLD include genetic predisposition, chronic inflammation, endothelial dysfunction, oxidative stress, and hemostatic alterations in the balance between pro- and anticoagulant factors [40]. In recent decades, single nucleotide polymorphisms in the genes that regulate lipid handling and secretion have been associated with fatty liver and its progressive forms.

## 5. Hypercoagulability in Patients with NAFLD

The presence of hemostatic alterations has been hypothesized to underlie both arterial cardiovascular injury (presence of arterial thrombosis (e.g., myocardial infarction, cerebrovascular stroke) and venous thromboembolism (e.g., increased rate of deep vein thrombosis, pulmonary embolism, and portal vein thrombosis)) [27]. However, the association of NAFLD with hypercoagulability, although surmised based on epidemiological observations, has not been widely evaluated.

The present review article aims to overview the causes and possible consequences of hypercoagulability in NAFLD to foster research work that may help to manage this important aspect in this group of patients.

### 5.1. Basic Tests of Coagulation in NAFLD

Typically, in patients with NAFLD, such basic tests of coagulation as the PT and APTT are within normal range or slightly prolonged, yet of negligible clinical significance [41]. This is not unexpected if one considers that these tests are not truly representative of the process that occurs in vivo (see above). In contrast, individual pro- and anticoagulants show variable degrees of abnormalities. Previous studies have shown that some procoagulant factors (namely factors VIII, IX, XI, and XII) are increased [42], whereas little information has been reported on the levels of naturally occurring anticoagulants.

In a relatively large study [41] of 113 patients with NAFLD, factor VIII (one of the most potent drivers of thrombin generation) increased from steatosis (99 U/dL, (71–150)) to metabolic cirrhosis (157 U/dL (64–232)), *p* < 0.001. Conversely, protein C (one of the most potent drivers of thrombin downregulation) decreased from steatosis (103 U/dL, (77–228)) to metabolic cirrhosis (77 U/dL (17–146)), *p* < 0.001. Consequently, the ratio of factor VIII/protein C, also taken as an index of hypercoagulability, increased from steatosis (0.96, (0.36–1.60)) or NASH to metabolic cirrhosis (2.05, (0.81–12.1)), *p* < 0.001 and was correlated with the ETP-TM ratio (rho = 0.543, *p* < 0.001). Antithrombin was reduced only in patients with metabolic cirrhosis (patients vs. controls, 78 U/dL, (33–123) vs. 109 U/dL (74–140)), *p* < 0.001).

### 5.2. Thrombin Generation in NAFLD

Thrombin generation expressed as the ETP-TM ratio increased from controls (0.57 (0.11–0.89)) to steatosis or NASH (0.72 (0.33–0.86)) and metabolic cirrhosis (0.80 (0.57–0.95)), (*p* < 0.001), the latter being comparable to that observed for alcoholic/viral cirrhosis (0.80 (0.57–0.95) vs. 0.80 (0.44–0.96)) taken as positive controls [41]. These results provide evidence that a state of hypercoagulability does exist in patients with NAFLD and that it progresses with the severity of the disease, being relatively low in steatosis and dramatically high in metabolic cirrhosis where it is similar to that observed in a group of patients with viral/alcoholic cirrhosis taken as positive controls. Similar results were obtained upon investigating the same cohort of patients by means of an additional procedure for thrombin generation, in which TM was substituted with Protac^®^, a snake venom, non-physiological activator, which shares the same protein C-activating properties as TM [41]. Interestingly, these values were also comparable in a subgroup of patients with less severe cirrhosis (Child A) of both etiologies.

**Figure 4:**
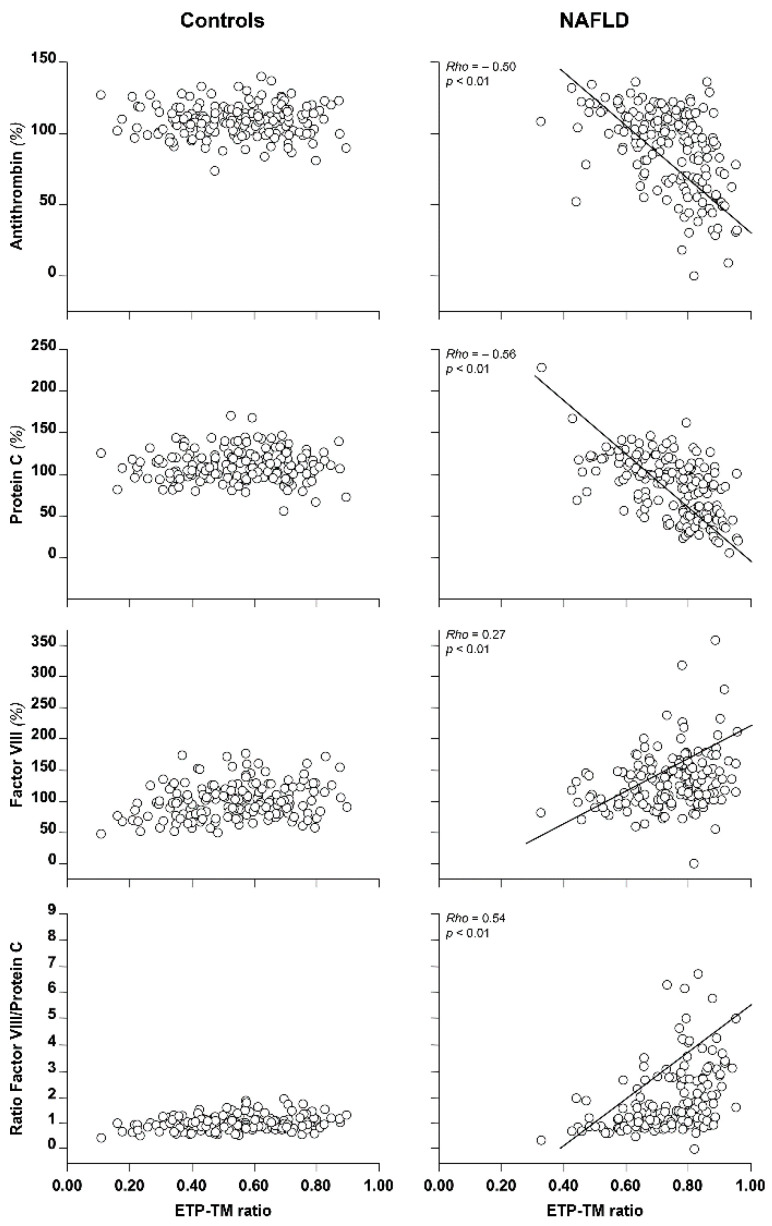
Correlation between coagulation parameters and the ETP-TM ratio in patients with NAFLD and health subjects. The ETP-TM ratio represents the ratio of endogenous thrombin potential (ETP) measured in the presence/absence of thrombomodulin (TM). Adapted with permission from ref. [41]. Copyright 2014 Elsevier (Amsterdam, The Netherlands).

Additional evidence that strengthens the relationship between the ETP-TM ratio and hypercoagulability in NAFLD is shown in Figure 4. Patients with NAFLD showed a significant inverse correlation of the ETP-TM ratio vs. antithrombin (rho = −0.50, *p* < 0.01) or protein C (rho = −0.56, *p* < 0.01), and a direct correlation of the ETP-TM ratio vs. factor VIII (rho = 0.27, *p* < 0.01) or vs. the ratio of factor VIII/protein C (rho = 0.54, *p* < 0.01). Furthermore, the ETP-TM ratio was directly correlated with the body mass index (rho = 0.26, *p* < 0.01), which is considered to be a generic risk factor for hypercoagulability. Interestingly, the above correlations were not evident in the control group (see Figure 4), suggesting that the ETP-TM ratio may be considered to be a reliable candidate laboratory tool for assessing hypercoagulability in patients with NAFLD. Additional evidence that supports the concept that hypercoagulability may play a role as a possible contributor to the risk of cardiovascular disease in NAFLD is the observation that patients with a ETP-TM ratio higher than the median value of controls had a significantly high risk of metabolic syndrome, high media-intima thickness, fibrosis, steatosis, or lobular inflammation [41], all typical features of NAFLD. Finally, additional contributors supporting the concept of hypercoagulability is the occurrence in NAFLD of increased NETs levels [43] and microvesicles [44], which, through dissemination into the circulation of procoagulant activity, may trigger coagulation and, consequently, thrombin generation. Inflammation, typically observed in the more severe form of NAFLD, namely NASH, may also trigger coagulation and thrombin generation through the release of proinflammatory mediators (e.g., IL-1, IL-6, and TNFα) [45]. Although the relationship between microvesicles, NETs, or inflammation and thrombin generation in NAFLD has not yet been directly explored, analogy with other diseases such as type II diabetes [12] and others [15] and the lack of correlation between the ETP-TM ratio and the individual components of coagulation observed in heathy controls (see Figure 4) support the concept of a causal relationship in patients with NAFLD.

More recently, Potze et al. [46] investigated a cohort of patients with NAFLD and concluded that hemostasis (i.e., platelets, coagulation, and fibrinolysis) was rebalanced and that there were no biochemical signs of hypercoagulability as measured by thrombin generation, despite elevated factor VIII, von Willebrand factor (another index of procoagulant imbalance), and reduced protein C reported in their cohort.

There are various explanations for the apparent contrasting conclusions reported by the two studies. First, the sample size was considerably different (n = 113 (ours) vs. n = 68 (Potze et al. study)). Therefore, it is possible that the latter study was underpowered to show statistical significance. In fact, the ETP-TM ratio in the Potze study (they called it TM-SR) tended to increase in patients with NASH cirrhosis as compared with controls, without reaching statistical significance. Second, Potze et al. [46] reported unusually high factor VIII in their population of controls (median, 144 U/dL vs. expected 100 U/dL). Therefore, it is possible that the selected control population possessed a state of hypercoagulability, as shown by high factor VIII that could have masked that observed for NAFLD. Third, the thrombin generation procedures in the two studies were not necessarily comparable. For example, Potze et al. [46] used tissue factor and negatively charged phospholipids at concentrations that were four times higher than those used in our study. The concentrations of tissue factor and negatively charged phospholipids are among the most important determinants of the sensitivity of thrombin generation parameters to detect hypo- or hypercoagulability [10]. Finally, Potze et al. [46] did not report on the type/concentrations of TM, another important determinant of the ETP-TM ratio. All in all, the above circumstances may well explain the contrasting results obtained in the two studies.

In conclusion, the experimental results of our study as well as the growing body of epidemiological observations, support the concept that NAFLD is associated with a state of plasma hypercoagulability, which alone or (more likely) in combination with other circumstantial risk factors may contribute to the progression of the disease from steatosis to the most severe form of metabolic cirrhosis, atherosclerosis, and the increased risk of cardiovascular diseases, which are typically observed in these patients.

## 6. Hypercoagulability and Anticoagulation in NAFLD

Although data in humans have consistently demonstrated the presence of a procoagulant imbalance in NAFLD, to date, few observations are available on the effect of anticoagulant therapy on hypercoagulability and, more generally, on the clinical outcome of fatty liver. On the contrary, interesting results have been observed in preclinical studies with animal models. Dabigatran, a direct thrombin inhibitor, has shown a protective effect on hepatic fibrin deposition, inflammation, and hepatocellular damage in mice treated with high-fat diets [47]. Furthermore, the activity of dabigatran has been related to a reduction in high-fat diet-induced weight gain [48]. Interestingly, possible interactions among anticoagulant activity, weight gain/obesity, and NAFLD were recently reported in humans by Wen et al. [49]. Lower average warfarin daily dosage and shorter time in the therapeutic range were found in patients diagnosed with NAFLD/NASH [49]. Notwithstanding, this anticoagulation in patients with NAFLD has been poorly investigated.

In addition to vitamin K antagonists (VKA), other drugs such as low molecular weight heparin (LMWH) or direct oral anticoagulants (DOAC), widely used in the general population to prevent venous thromboembolism and/or systemic embolism/ischemic stroke in patients with atrial fibrillation, could be potentially used in patients with NAFLD as a therapeutic strategy aimed to slow down the progression to liver fibrosis, typically observed in these patients [50,51,52]. For example, experimental observations in animal models have shown that coagulation modulated murine hepatic fibrogenesis and several clinical and preclinical studies have demonstrated a link between fibrogenesis and hyperactivation of hemostasis [53]. Specifically, it has been shown that the progression of liver fibrosis is mediated by hypercoagulability, which, in turn, can modulate various aspects of organ fibrogenesis through thrombin and the family of protease activated receptors (PAR-1/2), which are potent direct effectors of stellate cell activation and fibrosis. The above processes can be slowed down in animal models by the administration of LMWH, VKA, or DOAC, leading to downregulation of thrombin production (reviewed in [54]). Taken together, these observations suggest that anticoagulation could be useful for preventing/treating cardiovascular diseases in patients with NAFLD and also for preventing/treating progression to liver fibrosis.

## 7. Hypercoagulability and Statins in NAFLD

Statins are widely used as cardioprotective drugs in patients with heightened cardiovascular risk. Furthermore, recent nationwide cohort and nested case-control studies have shown that statin use in the general population was associated with reduced venous thromboembolism recurrence [55]. However, although considered to be relatively safe by guidelines [56,57,58,59,60], statins are not widely used to treat NAFLD. Indeed, there are animal studies and post hoc small clinical trials in humans that have shown some beneficial effects of statins in improving liver histology and, most importantly, risk reduction of cardiovascular diseases (reviewed in [57]). A recent relatively large nationwide nested case-control study investigated the effect of statins on the development and progression of NAFLD and concluded that statin use decreased the risk of NAFLD occurrence and the risk of liver fibrosis once NAFLD had developed [60]. Interestingly, statins seem to mediate their cardioprotective beneficial effect in patients with familial hypercholesterolemia, at least in part, by downregulating thrombin production [61]. The beneficial effect of statins on thrombin generation is probably mediated through a reduction in procoagulant factors such as factor VIII [62] or other unknown mechanisms.

## 8. The Value of Measuring Thrombin Generation in NAFLD

All in all, monitoring thrombin generation in patients with NAFLD could be a new laboratory tool for gaining insights into the pathophysiology of the disease and for helping to improve its treatment. Clinical trials with prospective design are, however, needed to establish conclusively any cause-effect relationship. In this respect, the above observations suggest that thrombin generation, expressed as the ETP-TM ratio, may be considered to be a reliable candidate laboratory tool for assessing, by means of a single procedure, the extent of hypercoagulability in patients with NAFLD. Specifically, thrombin generation expressed as the ETP-TM ratio could be helpful not only to define the state of hypercoagulability in this category of patients, but also as a reliable index parameter to assist organizing clinical trials needed to establish the treatment of choice (e.g., anticoagulation, use of statins, etc.) that may help reduce hypercoagulability and, hence, the sequel of adverse events known to burden NAFLD, such as the high risk of cardiovascular diseases, and progression to atherosclerosis and liver fibrosis. In this respect, based on pathophysiological considerations, in the future, anticoagulation and/or statin use could be attractive therapeutic strategies in patients with NAFLD.

## 9. Concluding Remarks

NAFLD is the most common chronic liver disease in Western countries, and it is anticipated that it could become even more prevalent in the general population in parallel with an increase in the prevalence of metabolic diseases that are closely associated with NAFLD (e.g., obesity, type II diabetes, dyslipidemia, and arterial hypertension). Therefore, NAFLD is a formidable burden for health systems across the world. In addition to liver impairment, NAFLD is associated with cardiovascular diseases (these being the most important causes of death in this condition), including atrial fibrillation, atherosclerosis progression, and venous thromboembolism that are all plausibly associated with hypercoagulability, which may, in turn, develop as a consequence of an imbalance of pro- vs. anticoagulants and the presence of such molecular species as procoagulant microvesicles, NETs, and inflammation that are associated with NAFLD. In addition, all these conditions may require anticoagulant therapy. The assessment of hypercoagulability by means of thrombin generation, a global procedure that mimics the coagulation process occurring in vivo much better than any other coagulation test, may be considered to be a candidate laboratory tool for assessing, with a single procedure, the balance of coagulation in NAFLD. In addition to defining the state of hypercoagulability, the assessment of thrombin generation could also be used to investigate patients with NAFLD enrolled in clinical trials, with the aim of determining the best approach (therapeutic and/or lifestyle change) for minimizing hypercoagulability and the risk of atherosclerosis progression and venous thrombosis in this condition.

## Data Availability

Not applicable as this is a review of the published literature.
